# An unexpected role for a glutamate receptor

**DOI:** 10.1126/science.adm6771

**Published:** 2023-12-21

**Authors:** Ian D. Coombs, Mark Farrant

**Affiliations:** Department of Neuroscience, Physiology, and Pharmacology, University College London, Gower Street, London WC1E 6BT, UK

## Abstract

Ion channels activated by the neurotransmitter glutamate underlie excitatory signaling between neurons in the brain. In mammals, these ionotropic glutamate receptors (iGluRs) belong to three families formed from GluA, GluN, and GluK subunits, respectively: the α-amino-3-hydroxy-5-methyl-4-isoxazolepropionic acid (AMPA), *N*-methyl-D-aspartate (NMDA), and kainate receptors (1). By contrast, receptors of a fourth homologous iGluR family—the δ or GluD receptors—do not respond to glutamate. Despite sharing a similar architecture with other iGluRs, including a transmembrane pore, the question of whether GluD receptors pass current has been controversial (2, 3). Instead, they are best known as synaptic organizing proteins (2, 4). On page 1389 of this issue, Piot et al. (5) show that one GluD family member, GluD1, can bind the inhibitory neurotransmitter γ-aminobutyric acid (GABA) and trigger potentiation of GABA-mediated synaptic currents. This challenges the dogmatic distinction between glutamate and GABA receptors and identifies GluD1 as a regulator of inhibitory signaling.

The GluD family members, GluD1 and GluD2, can both be found at excitatory synapses. They connect, through adaptor proteins of the cerebellin family, to presynaptic adhesion molecules of the neurexin family, forming trans-synaptic complexes that play key roles in synapse assembly and activity-driven synaptic modifications (6). These roles were first identified for GluD2 at synapses made by granule cell parallel fibers onto Purkinje cells in the developing cerebellum, where the binding of D-serine, released by Bergman glia, enables long-term depression of excitatory transmission by facilitating AMPA receptor internalization (7). GluD1 is similarly implicated in trans-synaptic interactions that have been shown to influence the formation of excitatory synapses (8) and the balance of AMPA receptor– and NMDA receptor–mediated signaling (9). However, distinct among iGluRs, GluD1 has also been found at GABA-releasing inhibitory synapses, specifically, at those between somatostatin positive interneurons and cortical pyramidal neurons. GluD1 interactions with cerebellin and neurexin, together with the binding of D-serine or glycine, are proposed to trigger intracellular signals that regulate the assembly of the inhibitory postsynapse (10).

Prompted by these findings, Piot et al. investigated the action of GABA on GluD1 receptors. Using recombinant receptors expressed in African clawed frog (*Xenopus laevis*) oocytes, they recorded currents from constitutively open GluD1 and GluD2 “Lurcher” mutants (11). The authors found that the GluD1 currents were enhanced by both D-serine and GABA, which competed for the same binding site. GABA was as efficacious as D-serine, albeit with much lower potency. By contrast, GluD2 currents were inhibited by D-serine but unaffected by GABA, hinting at multiple functional differences between GluD1 and GluD2, at least in their Lurcher forms. Piot et al. visualized GABA binding to GluD1 using x-ray crystallography of isolated GluD1 ligand-binding domains, which established that key molecular determinants of the binding are shared by GABA and D-serine, consistent with the competitive actions observed. Of critical importance for their subsequent experiments, the authors also identified point mutations in GluD1 that were able to abolish GABA binding while leaving D-serine binding largely intact.

To address the question of whether GABA could signal through wild-type (non-Lurcher) GluD1 receptors at synapses, Piot et al. recorded from CA1 pyramidal neurons in acute hippocampal slices from mice and electrically stimulated the release of GABA from neurons in the stratum lacunosum-moleculare (where the dendrites of CA1 pyramidal neurons are located), which is a region of intense GluD1 expression. Bursts of high-frequency synaptic stimulation, or application of D-serine, enhanced the amplitude of inhibitory postsynaptic currents (IPSCs) mediated by type A GABA (GABA_A_) receptors (see the figure). These effects were occluded by short hairpin RNA–based downregulation of GluD1 expression. Moreover, by introducing GluD1 mutants that lacked specific signaling capabilities, the authors showed that GABA binding and cerebellin interactions were required for the enhancement of IPSC amplitude but the passage of ions through GluD1 channels was not.

It remains to be determined which of the four isoforms of cerebellin (4) participate in the inhibitory plasticity described by Piot et al. and whether this “non-ionotropic” effect of GluD1 requires cerebellin to be engaged with presynaptic neurexins. This is a key question. GABAergic interneurons are famously heterogeneous, and such interactions might be expected to determine the cellular specificity of the plasticity. Multiple distinct interneuron subtypes are found in, or bordering, the stratum lacunosum-moleculare, prominent among which are neurogliaform cells (12). These form atypical synapses and, unlike most other interneurons, their activation results in prolonged GABA elevations that reach relatively low peak concentrations. Given this, the apparent low affinity of GluD1 for GABA is particularly intriguing.

How has the iGluR gating machinery been adapted by GluD1 to produce an apparently non-ionotropic effect? Of note, calcium ions greatly decrease the potency of D-serine at GluD2-Lurcher receptors by stabilizing the receptor ligand-binding domains in a dimeric conformation (13), likely dictating whether ligand binding ultimately engages gating- or desensitization-like states. The GluD1 ligand binding domain structures obtained by Piot et al. contain calcium ions bound at the dimer interface; whether the functional influence of these ions is similar to that seen with GluD2, and how this affects the synaptic action of GABA on GluD1, are important issues. Some of these questions might be addressed by using cryo–electron microscopy to visualize intact GluD1 receptors in the presence and absence of GABA and calcium ions.

Beyond the immediate mechanics of GluD1 “activation”, it is not known which downstream effector proteins and signaling pathways are ultimately key to IPSC potentiation nor how this potentiation interacts with other forms of inhibitory plasticity. The low affinity of GABA, and, therefore, the presumed brevity of the GluD1 signal (even during high-frequency synaptic stimulation), suggests a tight linkage between GluD1 and its effector. An unbiased proteomic screen previously identified several potential GluD1 interacting proteins 10), but their roles in the newly described GABA-induced plasticity remain to be determined. It is interesting to note the recent recognition that alternative splicing can give rise to GluD1 isoforms with different carboxy-terminal cytoplasmic tails (14). This raises the possibility of different cohorts of GluD1 binding partners and thus the involvement of different signaling pathways that are potentially dependent on the type of synapse or developmental stage.

In humans, numerous variants of the gene encoding GluD1 (*GRID1*) have been identified. These copy number and missense variants are associated with several neurological conditions, including schizophrenia, autism, intellectual disability, and seizures (15). Whether the disruption of GluD1-dependent inhibitory plasticity plays any role in the effects of disease-associated GRID1 variation is an important question for future study.

## Figures and Tables

**Figure 1:**
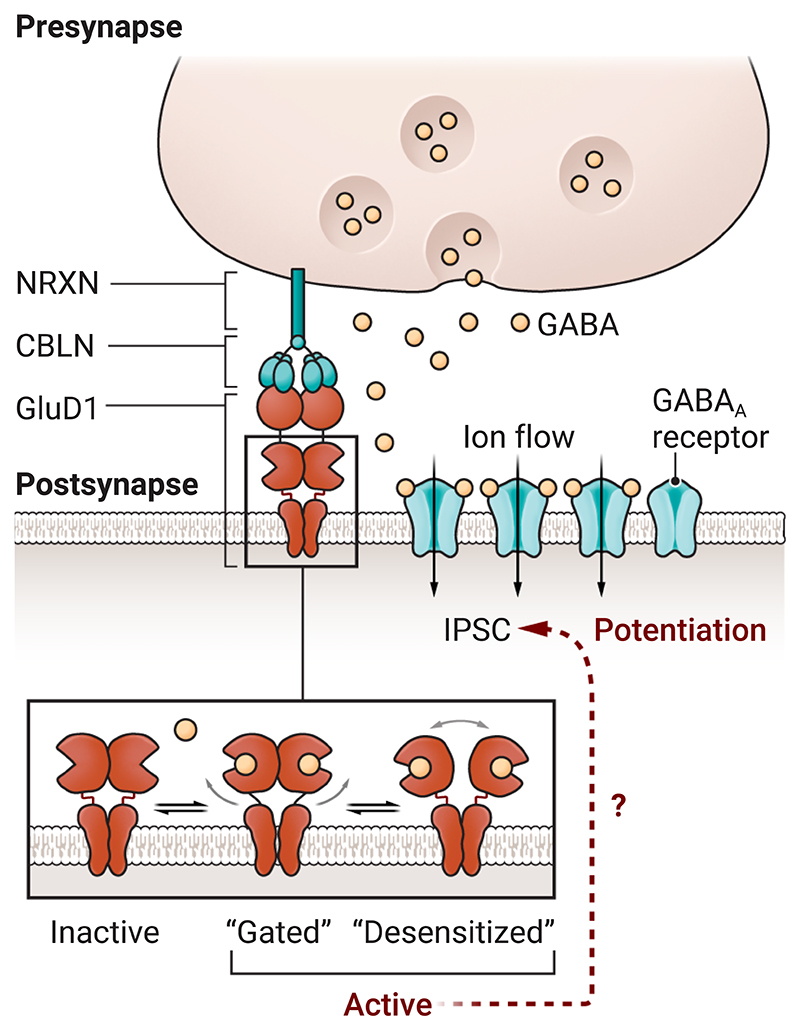
A new mediator of inhibitory plasticity. GluD1 receptors form trans-synaptic complexes that include cerebellins (CBLN) and neurexin (NRXN). At an inhibitory hippocampal synapse, GluD1 can bind γ-aminobutyric acid (GABA), causing the potentiation of type A GABA (GABA_A_) receptor–mediated inhibitory postsynaptic currents (IPSCs). When GluD1 binds GABA (two of the four subunits are shown), it likely undergoes structural changes akin to those leading to gated and desensitized states in other ionotropic glutamate receptors, but the mechanism that leads to potentiated IPSCs is unknown.
